# Recurrent intrahepatic cholestasis of pregnancy incidence and risk factors: A retrospective cohort study

**DOI:** 10.1002/pmf2.70055

**Published:** 2025-06-13

**Authors:** Inbar Lifshitz, Geffen Kleinstern, Roy Zigron, Amihai Rottenstreich

**Affiliations:** ^1^ Faculty of Medicine Hadassah‐Hebrew University Medical Center Jerusalem Israel; ^2^ School of Public Health University of Haifa Haifa Israel; ^3^ Department of Obstetrics and Gynecology Hadassah‐Hebrew University Medical Center Jerusalem Israel; ^4^ Department of Obstetrics and Gynecology E. Wolfson Medical Center Holon Israel; ^5^ School of Medicine Tel Aviv University Tel Aviv Israel

**Keywords:** bile acids, intrahepatic cholestasis of pregnancy, prediction, pregnancy, recurrence

## Abstract

**Objectives:**

The incidence of intrahepatic cholestasis of pregnancy (ICP) varies throughout the world and is influenced by genetic, hormonal, and environmental factors. However, data are limited regarding the risk of ICP recurrence in subsequent pregnancies. We aimed to determine the incidence of recurrent ICP and its associated risk factors.

**Methods:**

A retrospective cohort study conducted at a university hospital during 2007–2023, including all pregnant individuals who underwent serum bile acids (BA) level testing due to suspected ICP. Among those diagnosed with ICP, records were reviewed to identify those with a subsequent gestation.

**Results:**

Of 640 individuals who met the inclusion criteria, 22.2% (*n* = 142) were diagnosed with ICP (fasting serum BA > 10 µmol/L) in the index pregnancy. Of them, 51 (35.9%) had a subsequent pregnancy. The rate of recurrent ICP in the subsequent pregnancy was 29.4% (15/51). Timing of ICP diagnosis did not differ significantly between the index and subsequent gestation (median 33 [IQR 30–36] vs. 34 [IQR 31–35] weeks, *p* = 0.90). In multivariable analysis, serum BA levels at the index pregnancy above 40 µmol/L were the only independent factor associated with ICP recurrence.

**Conclusions:**

Recurrence of ICP is common, occurring in over one‐fourth of patients with a history of ICP. BA levels at the first pregnancy in which ICP was diagnosed were found as the only independent factor associated with the occurrence of ICP in subsequent gestation.

**Abbreviations**
BA: bile acids
ICP: intrahepatic cholestasis of pregnancy

## 1 INTRODUCTION

Intrahepatic cholestasis of pregnancy (ICP) is the most common liver disorder in pregnant patients [1, 2]. ICP usually manifests in the second and third trimesters of pregnancy with associated maternal pruritus [3]. While maternal pregnancy outcomes are generally favorable, ICP is associated with various adverse perinatal outcomes.

ICP is characterized by increased concentrations of serum bile acids (BA), which currently serve as the most sensitive marker for the diagnosis of this condition [4, 5, 6]. Most importantly, BA levels not only serve for the diagnosis of ICP, but also correlate with the severity of this disorder [7, 8, 9, 10]. It has been previously demonstrated that higher BA levels are associated with increased likelihood of experiencing adverse outcomes, including preterm birth, meconium‐stained amniotic fluid, and stillbirth [7, 8, 9, 10].

A key concern for patients affected by ICP is risk of recurrence in future pregnancies. There is limited information on the risk of recurrent ICP in subsequent gestations, with prior studies reporting highly variable recurrence rates ranging between 31% and 92% [11, 12, 13, 14, 15]. Moreover, the specific predictors of recurrence, including the level of BA levels, are largely unknown. Given the paucity of literature and the high clinical relevance, we aimed to evaluate the recurrence rate of ICP and the associated risk factors, including the level of BA levels in the first ICP‐affected pregnancy.

## 2 MATERIALS AND METHODS

### 2.1 Study population

This is a retrospective cohort study conducted at a university‐affiliated tertiary hospital during 2007–2023. The center serves large, heavily populated urban areas as well as rural areas, and treats a heterogeneous population with over 10,000 deliveries per year. The study cohort included all pregnant individuals who underwent serum BA testing due to new‐onset pruritus. The timeframe was selected based on the availability of BA‐level testing, which became standardized at our center from 2007 onward, ensuring consistency and accuracy in measurement across the study period.

Patients were identified from the electronic database of the general laboratory in our center. ICP was diagnosed throughout the study period based on serum BA levels above 10 µmol/L [5, 6]. Records of pregnant patients diagnosed with ICP were reviewed to identify those with a subsequent gestation. Exclusion criteria included pre‐existing hepato‐biliary disease, dermatologic conditions, allergic drug reaction or drug‐induced liver injury, suspected acute fatty liver of pregnancy, or hemolysis or HELLP (hemolysis, elevated liver enzymes and low platelet) syndrome.

### 2.2 Data collection

The electronic databases of the laboratory and obstetric units in our medical centers were used for the purpose of this study. We retrieved maternal hospital admission records (including both inpatient and outpatient encounters), discharge summaries, and laboratory results from the aforementioned databases by an electronic query. Maternal and pregnancy characteristics, as well as laboratory results, were extracted. To confirm the accuracy of data, records were also manually reviewed by two independent investigators, and disagreement was resolved by consulting a third reviewer.

### 2.3 Laboratory investigations

Serum BA testing was performed using a commercially available enzymatic assay (Diazyme Total Bile Acids Assay Kit, Diazyme Laboratories) at the Clinical Biochemical laboratory of our center. During the study period, serum BA‐level determination was made following a 12–14 h fast. All patients were sampled prior to commencing ursodeoxycholic acid. Elevated serum BA levels were confirmed by repeated testing. Serial BA evaluations were performed based on clinical judgment. If serial BA measurements were available in the same patient, the highest measurement was recorded.

### 2.4 Statistical analysis

Among patients diagnosed with ICP who had a subsequent gestation, maternal and pregnancy characteristics and laboratory results were analyzed and compared between those with and without recurrent ICP. Characteristics of patients are described as proportions for categorical variables and as medians and interquartile ranges (IQR) for continuous variables without a normal distribution. Multivariate logistic regression analysis was used to adjust for potential confounding factors for recurrent ICP, reported as odds ratio (OR) and 95% confidence interval (CI); potential associated factors included those identified in the univariate analysis (*p* value < 0.05) and known clinically relevant parameters (maternal age and gestational age at the time of testing). Significance was assessed by the Chi‐square test and Fisher's exact test for categorical variables. The Student *t*‐test was used for the analysis of continuous variables with normal distribution and the Mann–Whitney *U* test for analysis of continuous variables with skewed distribution. A two‐sided *p* value < 0.05 indicated statistical significance. The data were analyzed using Software Package for Statistics and Simulation (IBM SPSS version 27, IBM Corp).

### 2.5 Ethical approval

The study was approved by the Human Investigation Review Board (IRB approval number: HMO ‐ 0107‐ 19).

## 3 RESULTS

**FIGURE 1:**
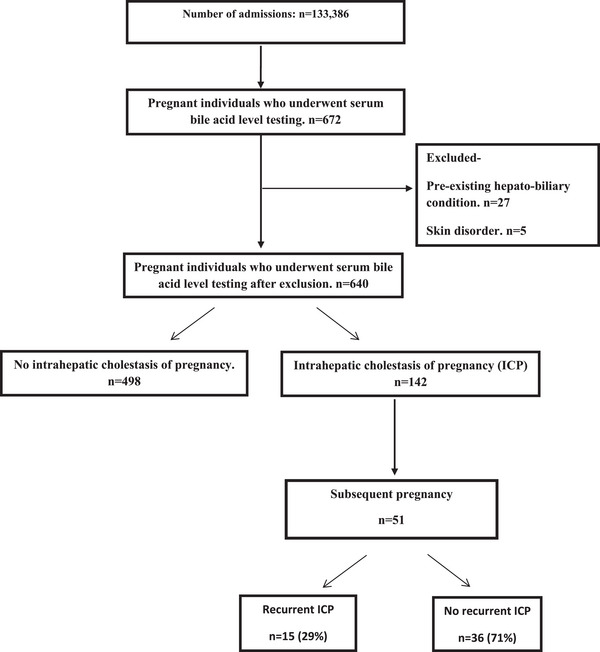
Schematic flow chart of patient inclusion in the study.

During the study period, 672 pregnant individuals underwent serum BA level testing at our center, with 640 (95%) meeting the inclusion criteria. Overall, 142 (22.2%) were diagnosed with ICP (fasting serum BA > 10 µmol/L) in the index pregnancy, including 115 (81.0%) patients with serum BA levels between 10 and 40 µmol/L, 22 (15.5%) with levels of 40–100 µmol/L, and 5 (3.5%) patients with levels higher than 100 µmol/L. Of these 142 patients, 51 (35.9%) had a subsequent gestation. A flow chart describing the establishment of the study cohort is shown in Figure 1.

Neither the serum BA levels at the index pregnancy (median 18.8 vs. 18.3 µmol/L, *p* = 0.93) nor the severity of ICP (serum BA levels categories: 10–40 µmol/L: 76.4% vs. 83.5%; 40–100µmol/L: 17.6% vs. 14.3%; > 100 µmol/L: 5.9% vs. 2.2%, *p* = 0.43) differed between patients with and without a subsequent pregnancy.

The median interpregnancy interval was 3 [IQR 2–4] years. Among those with a subsequent gestation (*n* = 51), 15 (29.4%) experienced recurrent ICP (Figure 1). In those with recurrent ICP (*n* = 15), serum BA levels at the time of ICP recurrence were between 10 and 40 µmol/L in 13 (86.7%) patients, with levels of 40–100 µmol/L in the remaining two (13.3%) patients, with a median level of 17.6 µmol/L. Timing of BA measurement did not significantly differ between the index and subsequent gestation (median 34 [IQR 31–35] vs. 33 [IQR 30–36] weeks, *p* = 0.90).

Characteristics of those with and without recurrent ICP are summarized in Table 1. Serum BA levels at the index pregnancy in those who experienced recurrence of ICP in the subsequent gestation were significantly higher than those without recurrent ICP (median 30.0 vs. 16.7 µmol/L, *p* = 0.04). The rate of recurrent ICP was significantly higher in those with BA levels above 40 µmol/L at the index pregnancy as compared to those with levels of 10–40 µmol/L (7/12, 58.3% vs. 8/39, 20.5%, OR [95% CI]: 5.43 (1.36–21.70), *p* = 0.02). Other maternal and obstetric characteristics did not differ between those with and without recurrence of ICP (Table 1).

**TABLE 1:** Characteristics of patients with intrahepatic cholestasis of pregnancy who had a subsequent pregnancy.

Characteristics	No recurrent ICP^a^ *n* = 36	Recurrent ICP^a^ *n* = 15	*p* value
Characteristics of index pregnancy			
Maternal age (years)	27 [25–29] (27)	26 [22–31] (27)	0.79
Gravity	1 [1–3] (2)	2 [1–4] (3)	0.49
Parity	0 [0–2] (1)	1 [0–2] (1)	0.50
Nulliparous (%)	21 (58.3%)	7 (46.7%)	0.54
ART conceived pregnancy (%)	6 (16.7%)	2 (13.3%)	0.99
Twin gestation (%)	6 (16.7%)	1 (6.7%)	0.43
Gestational age at bile acids testing (weeks)	34 [32–36] (33)	34 [31–35] (33)	0.77
Gestational diabetes mellitus	5 (13.9%)	1 (6.7%)	0.65
Gestational hypertensive disorders	3 (8.3%)	1 (6.7%)	0.99
Bile acids (µmol/L)	16.7 [12.0–27.9] (23.7)	30.0 [14.7–80.0] (53.0)	0.04
<40 µmol/L	31 (86.1%)	8 (53.3%)	0.03
≥40 µmol/L	5 (13.9%)	7 (46.7%)	
Gestational age at delivery < 37 weeks (%)	28 (77.7%)	12 (80.0%)	0.99
Characteristics of subsequent pregnancy			
Maternal age (years)	31 [28–32] (30)	30 [25–33] (30)	0.62
ART conceived pregnancy (%)	6 (16.7%)	1 (6.7%)	0.66
Twin gestation (%)	2 (5.6%)	1 (6.7%)	0.99
Gestational age at bile acids testing (weeks)	32 [29–35] (31)	33 [30–36] (33)	0.31

*Note*: All continuous variables are expressed as medians [interquartile range] (mean).

Abbreviations: ART, assisted reproductive technology; ICP, Intrahepatic cholestasis of pregnancy.

^a^ ICP defined as bile acid level above 10 µmol/L.

Among those with a subsequent gestation (*n* = 51), one patient had experienced stillbirth in the index pregnancy, occurring at 35 weeks gestation with BA level of 92 µmol/L. In her subsequent pregnancy, she experienced recurrent ICP with BA level of 34 µmol/L, and following induction of labor at 35 weeks of gestation, delivered a live infant.

In a multivariable logistic regression analysis, serum BA level above 40 µmol/L at the index pregnancy (aOR [95% CI]:7.62 (1.56, 37.16) was the only independent factor associated with recurrence of ICP in subsequent gestation (Table 2).

**TABLE 2:** Multivariate logistic regression analysis of factors associated with recurrent ICP.

Variable	aOR (95% CI)	*p* value
Maternal age	0.91 (0.77–1.09)	0.31
Gestational age at bile acids testing at subsequent pregnancy	1.06 (0.89–1.27)	0.52
Bile acids ≥ 40 µmol/L at index pregnancy	7.62 (1.56–37.16)	0.01

*Note*: Adjusted for maternal age, gestational age at bile acids testing, bile acids levels at index pregnancy.

Abbreviation: aOR, adjusted odds ratio; CI, confidence interval; ICP, intrahepatic cholestasis of pregnancy.

## 4 DISCUSSION

In this study, we aimed to examine the risk of recurrence of ICP in a subsequent pregnancy, and to identify factors associated with recurrent ICP. Recurrence of ICP was observed in 29% of patients. History of severe ICP (serum BA above 40 µmol/L) was the only independent factor associated with recurrence.

The incidence of ICP varies throughout the world and is influenced by genetic, hormonal, and environmental factors [16, 17, 18, 19, 20, 21]. A higher incidence of ICP was reported in association with chronic liver diseases, multifetal pregnancy, family history of ICP, and advanced maternal age [16, 17, 22]. Moreover, the incidence of ICP can be influenced by the time of BA measurements as BA levels rise after meals. However, there is currently no consensus as to whether BA testing should be performed fasting or postprandially [16]. During the study period, BA measurements were universally carried at fasting, and may at least partially account for the relatively low incidence of ICP.

Patients with a history of ICP in prior gestation are at risk for recurrence, however, the degree of risk is largely unknown. The inconsistency in reported recurrence rates in the literature may be attributed to differences in study populations, diagnostic criteria, and most importantly, to the absence of systematic bile acid testing in prior studies [11, 12, 13, 14, 15]. In the current study, we report a 29% rate of recurrent ICP based on serum BA evaluation, which was carried in all patients, underscoring the validity of our findings.

In addition to the high variability in reported rates of ICP recurrence, there is limited information on the predictors of recurrence in subsequent gestation. We found that serum BA above 40 µmol/L at the index pregnancy was the only predictor associated with recurrence, with 58% recurrence rate in this subset of patients. This concurs with the findings of Wang et al. who reported a recurrence rate of 86% in those with a history of severe ICP [15]. While patients with a history of liver disease were excluded in our study, in their cohort chronic liver disease was evident in a substantial proportion of patients, mostly due to hepatitis B virus (HBV) with associated early onset of ICP, limiting the generalizability of their results.

Serum BA level testing is currently the gold standard for the diagnosis of ICP [4, 5, 6]. Nevertheless, measurement of BA levels is not performed in many institutions, and in many others, it is not available 24/7 with a relatively long turnaround time. Moreover, we previously demonstrated that various routine liver tests have low positive predictive value in the diagnosis of ICP [23], questioning the practice of offering induction of labor in pregnant individuals with suspected ICP solely based on impaired liver tests in the absence of confirmatory serum BA levels testing. The current study findings further underscore the importance of serum BA measurement not only to confirm the diagnosis of ICP, but also to predict the risk of recurrence.

### 4.1 Strengths and limitations

Our study has several potential limitations. The retrospective design of this study raises the possibility of biases inherent to such investigations. In this regard, while we excluded those with prior history of pre‐existing liver disease, routine sonographic liver evaluation was not performed. Moreover, in the current cohort only a minority of patients had serum BA levels higher than 100 µmol/L, a value above which an adverse perinatal outcome is more likely to occur [6, 7]. Finally, the risk of recurrent ICP may be affected by other factors that could not be assessed in the current study, including genetic factors. Therefore, coupled with the strong geographic variation of ICP, the generalizability of our findings should be further confirmed in other populations.

## 5 CONCLUSIONS

In conclusion, in the current study, we were able to evaluate the risk of ICP recurrence in subsequent gestation based on BA measurement, providing a clearer estimate than previous reports. Furthermore, we identified bile acid levels above 40 µmol/L at the previous pregnancy as the sole independent predictor of recurrence. The current study findings may have important clinical implications and should be implemented in patient counseling. Further prospective studies are warranted to confirm our findings and better delineate the predictive factors of recurrent ICP.

## AUTHOR CONTRIBUTIONS

**Amihai Rottenstreich**: study design, data coordination, data analysis, manuscript writing. **Inbar Lifshitz**: study design, data coordination, data analysis. **Roy Zigron**: study design, data coordination, data analysis. **Geffen Kleinstern**: manuscript writing, data analysis.

## CONFLICT OF INTEREST STATEMENT

The authors declare no conflicts of interest.

## ETHICS STATEMENT

All procedures performed in studies involving human participants were in accordance with the ethical standards of the institutional and/or national research committee and with the 1964 Helsinki Declaration and its later amendments or comparable ethical standards. This study was approved by the local institutional review board (IRB approval number: HMO ‐ 0107‐ 19).

## CONSENT

Informed consent was waived by the local institutional review board (IRB approval number: HMO ‐ 0107‐ 19).
